# Complications of Prostate Cancer Treatment: Open Issues

**DOI:** 10.3390/jcm13113090

**Published:** 2024-05-24

**Authors:** Javier C. Angulo

**Affiliations:** 1 Clinical Department, Faculty of Biomedical Science, Universidad Europea, Carretera de Toledo, Km 12.500, Getafe, 28905 Madrid, Spain; javier.angulo@universidadeuroepa.es; Tel.: +34-699497569; 2 Department of Urology, Hospital Universitario de Getafe, Carretera de Toledo, Km 12.500, Getafe, 28905 Madrid, Spain

Unfortunately, prostate cancer treatment is not free of complications. Despite the success achieved in every treatment option including surgery, radiotherapy, focal therapy, hormone therapy and immunotherapy, patients with prostate cancer diagnosed at different stages are still at high risk of suffering treatment-related complications. Of course, such complications are more unlikely if prostate cancer has been diagnosed and treated early in a lower-risk scenario, i.e., when complications weigh more negatively in the quality of life related to life expectancy ratio. However, prostate cancer is not always a slow-progressing disease. Additionally, demographic-based predictions suggest that the number of new cases of prostate cancer will reach 35 million by 2050, bringing a huge economic burden and treatment-related complications over the coming decades [1].

Sometimes the complications are inherent to the treatment regime elected and somehow depend on the circumstances involved, such as experience and technical issues, but other times the complications depend on patient characteristics. In this sense, Farzat et al. (Contribution 1) present an interesting study in the Special Issue that confirms that the body mass index of a patient has an impact on the complications and readmission rate of robotic radical prostatectomy, and also that the effectiveness of surgery as determined by positive surgical margin rate. Additionally, increased BMI could be associated with adverse pathology risk [2]. Furthermore, nerve-sparing surgical procedures in patients with obesity are technically more difficult. Having said this, the study of Farzat et al. confirms that robot-assisted radical prostatectomy is a feasible and safe therapeutic procedure even in patients with obesity.

Post-prostatectomy stress urinary incontinence is one of the major complications that affect the quality of life of prostate cancer survivors. The artificial urinary sphincter (AUS) has been the typical solution for this group of patients, especially when urine loss is large and pelvic-floor rehabilitation techniques have provided insufficient postoperative improvement. This alternative was proposed in the 1970s [3]; however, it is also not free of complications that include urethral erosion, urethral atrophy and device malfunction. Taking this into account and with an improved knowledge of continence damage after prostate surgery, male retro-urethral slings have been developed in the last three decades as a less invasive alternative to treat a very high proportion of patients with less severe male incontinence after prostatectomy.

An interesting male sling is the adjustable trans-obturator male system (ATOMS; A.M.I. GmbH, Feldkirch, Austria) designed by Wilhelm Bauer in Vienna, with the first results published in 2012 [4]. Many clinical studies have been performed that confirm that this device, the ATOMS, is a reasonable alternative to the AUS when some residual sphincteric function remains and has the advantage that it does not require patient manipulation to urinate. The superiority of this system upon a retro-urethral trans-obturator mesh is that the compressive effect produced by the ATOMS on the bulbar urethra can be adjusted postoperatively, and that is not possible in a regular simple mesh. Therefore, the ATOMS can be used in most cases with mild-to-moderate stress incontinence after prostatectomy, and in selected cases with severe urine loss. Multicentric studies, prospective evaluation and meta-analysis confirm that this therapeutic option is effective and safe and, most importantly, allows satisfactory patient-reported outcomes. In fact, it is the second most frequently used alternative for post-prostatectomy incontinence in Europe [5].

However, the ATOMS is not free of complications itself. In relation to this, the article by Giammò and Ammirati (Contribution 2) presents the long-term data on device survival and complications after ATOMS implant. The article is of merit because it is based on a cohort of patients treated and followed in a single institution, with a median follow-up exceeding 5 years. Additionally, the discussion they present gives an interesting overview of the topic and compares data regarding ATOMS with other anti-incontinence adjustable devices currently available. According to Giammò and Ammirati, as many as 88% of ATOMS implants remain in place at 60 months, and this device’s durability is larger than the one reported for an AUS, a system at a higher need of surgical revision [6,7].

Dealing with this topic, Queissert et al. present another interesting article focused on revision surgery in patients with ATOMS (Contribution 3). This is a relatively infrequent situation but may be necessary when the device does not achieve optimal continence results or when major complications lead to device explant. A previous multicenter study performed on the subject revealed that approximately 8% of ATOMS need surgical revision at a mean follow up of 4 years, and the main causes for revision are persistence of incontinence and scrotal port erosion, one of the most fearful late complications [8]. The choices after the explant of an ATOMS can be a second ATOMS implant or an AUS. Noticeably, postoperative results in terms of urine loss and patient satisfaction favor repeat ATOMS [8]. The article of Queissert et al. is a case series based on four patients in which repositioning of the ATOMS is optimized by more careful proximal implantation of the device. However, it should be specified that this is not a “new” technique as it is based in the principles of the original surgical description of Bauer [4,9]. Also, it is the same device, the silicone-covered scrotal port (SSP) ATOMS, that is the only system currently available.

Having said that, Queissert et al. (Contribution 3) advocate for an implantation as proximal as possible in cases of repeat ATOMS surgery, but their assumption is based exclusively on the observation of several cases and not in a head-to-head comparison; therefore, it should be taken simply as a hypothesis. In fact, only in one of the four cases does postoperative urine loss become “zero”. A more serious comparative trial should be needed to declare if this modification has a real impact on the results without adding complications. In fact, opening the bulbospongiosus muscle and cutting the perineal central tendon is necessary to place the ATOMS cushion closer to the membranous urethra, which can be difficult and possibly unnecessary in all cases for an optimized second implant placement. The anatomy of the patient must also be taken into account and several factors such as the size of the ischiopubic bone arch and the length of the bulbar urethra, related to different degrees of obesity, determine whether the cushion is placed more proximal or more distal. These factors are subject to individual variability and are also determinants of the cushion placement after trans-obturator passage.

Therefore, the proximal placement of the device should avoid cushion balancing due to unequal or asymmetrical passage of the tunneler through the obturator muscle on both sides. In my opinion, these items are important to achieve optimal ATOMS implantation, and should be cared for not only in a second procedure but also in naïve cases. Small variations could have a big effect, not in every case, but in patients with more severe sphincteric damage, i.e., those in which optimal postoperative adjustment is fundamental. The lessons Queissert and his colleagues learnt from ATOMS revision surgery are still interesting observations to discuss.

A more troublesome scenario in terms of complications is the combination of radical prostatectomy and other disadvantageous clinical scenarios, such as combined radiotherapy, urethral stricture or devastated bladder neck. Three different contributions consider these situations after prostate cancer treatment in this Special Issue. Angulo et al (Contribution 4). have published a multi-institutional study including patients treated with ATOMS after radical prostatectomy combined with pelvic radiotherapy. This population of patients has a higher risk of incontinence and achieves worse results than patients treated with radical prostatectomy alone [10,11]. However, this article is useful to identify the population of patients treated with ATOMS at higher risks of achieving dryness. The combination of determinant factors of success is presented in a nomogram that is based on the combination of naïve continence surgery, urethral or bladder neck stricture and baseline incontinence severity. Using this nomogram, a patient treated with prostatectomy plus adjuvant radiation can be individually counselled with a personalized chance of success with an ATOMS.

Sterling et al. (Contribution 5) have presented a very interesting review on one of the most fearful complications of prostate cancer treatment: post-radiation urethral stricture. The review presents pathophysiology and the prevalence of this radiation-induced local complication. A global view of the tissular changes and molecular pathways involved in post-radiation stricture correlates with the surgical pitfalls and therapeutic options, which are also well presented and discussed. The management of this complicated situation ranges from conservative and endoscopic treatment to different modalities of urethroplasty, either using excision and primary anastomosis or buccal mucosa graft urethroplasties. In this sense, a series with dorsal buccal graft augmentation urethroplasty has been recently published by the same group with very satisfactory results in this complex scenario [12].

Another article in this Special Issue, also by Queissert et al. (Contribution 6) on behalf of the Debates on Male Incontinence (DOMINO) Project, evaluates the AUS AMS 800 (Boston Scientific, Marlborough, MA) for the irradiated patient with a devastated bladder outlet produced by a stricture in the area of the membranous urethra or bladder neck. It has already been stated that erosion and urethral atrophy with an AUS are more frequent after radiation [13,14]. The analysis of a retrospective cohort of 62 patients implanted with AUS after radiotherapy and previous treatment of membranous urethral stricture or bladder neck contracture (different treatment modalities including endoscopic treatments and open reconstruction) definitely confirms a lower effectiveness of AUS and higher revision rate. Several forms of urinary diversion and vesicostomy with bladder neck closure can be considered in this unfortunate clinical situation. In summary, many issues regarding postprostatectomy incontinence remain unsolved and can be the object of further investigations.
